# Oncologic and obstetric outcomes after conization for adenocarcinoma in situ or stage IA1 cervical cancer

**DOI:** 10.1038/s41598-020-75512-9

**Published:** 2020-11-16

**Authors:** Xiaoyu Wang, Yalan Bi, Huanwen Wu, Ming Wu, Lei Li

**Affiliations:** 1 Department of Obstetrics and Gynecology, Peking Union Medical College Hospital, Shuaifuyuan No. 1, Dongcheng District, Beijing, 100730 China; 2 Department of Pathology, Peking Union Medical College Hospital, Beijing, 100730 China; 3 Department of Dermatology, Beijing Tsinghua Changgung Hospital, Beijing, 102218 China

**Keywords:** Cancer therapy, Cervical cancer

## Abstract

This study aims to reveal the risk factors associated with recurrence or new-onset high-grade squamous intraepithelial lesions (HSILs) or more severe lesions (HSILs +) and analyze obstetrical outcomes in patients with adenocarcinoma in situ (AIS) or stage IA1 cervical cancer patients after conization. A retrospective cohort study was developed from January 1, 2002, and July 1, 2018, in a single center, where all patients with AIS or stage IA1 cervical cancer who accepted conization for primary surgery were reviewed and followed up until July 1, 2019, for the pathological findings of HSILs + and obstetric outcomes. Two hundred and seventeen patients were identified, including 114 cases of AIS, 76 cases of stage IA1 squamous cell carcinoma (SCC) and 27 cases of stage IA1 adenocarcinoma (ADC). A total of 88 (40.6%) patients had an intact uterus without radiotherapy. Five patients experienced HSIL+ recurrence. The cumulative 3-, 5- and 10-year incidence rates of HSILs + were 1.0%, 1.5% and 2.0%, respectively. No significant risk factors, including primary disease, margin status and hysterectomy, were associated with recurrence. Twenty (66.7%) of 30 patients who attempted pregnancy had 23 successful pregnancies, which result in 7 miscarriages, 16 live births and 5 preterm births. Age at conization was the only independent risk factor associated with pregnancy, live births and preterm births. In conclusion, conization is safe for young women with AIS, stage IA1 SCC and ADC who desire future fertility, and the associated HSIL recurrence rate is low. Increased age significantly lowered the conception or live birth rate.

## Introduction

Cervical cancer is one of most common causes of cancer-related deaths among women worldwide^1^ and in China^2^. Cervical cancer cases in China show an increased prevalence among young patients and at early stages^3^. Conservative, fertility-preserving surgical procedures have become the standard of care for women with low-risk, early-stage disease^4^. In patients with stage IA1 squamous cell carcinoma (SCC) or adenocarcinoma (ADC), the risk of lymph node metastasis is less than 1%^5–7^, the mean age of patients with AIS is 37 years^8^, and 40% of women with IA1 cervical cancer are between 25 and 49 years old^9^. Hence, for stage IA1 patients without lymphovascular space invasion (LVSI), conization and simple extrafascial hysterectomy can be performed for those wishing or not wishing to preserve fertility, respectively^10–12^. The subsequent treatment is based on margin status and surveillance results^10^. ADC in situ (AIS) and endocervical ADC (or the usual type) belong to human papillomavirus (HPV)-associated ADC according to the International Endocervical Adenocarcinoma Criteria and Classification^13^^.^ Recurrence and residual disease have been reported in up to 50% of cases of AIS^8^. As ADC is likely to develop metastasis outside the pelvis and is associated with a significantly poor prognosis, radical treatment is recommended^14^. However, recent studies have also demonstrated that the clinical outcomes of women with microinvasive ADC are comparable to those of their SCC counterparts and can be safely managed with fertility-preserving procedures^15–17^.

However, little is known about the potential risk factors influencing the oncologic and/or obstetric outcomes of AIS, stage IA1 SCC and ADC. Many previous studies included only small numbers of women and had heterogeneous designs. Most studies were retrospective and therefore had a high risk of bias^18^. Most of these studies emphasized the association between the surgical procedure and preterm birth. Accurate conception and live birth rates after fertility-sparing surgeries remain unclear. For patients with AIS and stage IA1 SCC or ADC, whether hysterectomy or a negative margin status guarantees oncologic safety is still debatable. Actionable factors that may promote a high conception rate have seldom been explored.

The current study aimed to explore oncologic and obstetric outcomes in a large cohort of patients with conization for AIS or stage IA1 cervical carcinoma.

## Materials and methods

### Ethical approval

This is a retrospective cohort study implemented in a tertiary teaching hospital. The Institutional Review Board of Peking Union Medical College Hospital approved the study (No. S-K777). The registration number is NCT03961178 (*clinicaltrials.gov*). Written informed consent was obtained from all participants in the study. All procedures involving human participants were performed in accordance with the ethical standards of the institutional and/or national research committee and with the 1964 *Declaration of Helsinki* and its later amendments or comparable ethical standards.

### Study design and patient enrollment

Patients were derived from the study center between January 1, 2002, and July 1, 2018. Only patients with histological confirmation of AIS, stage IA1 (International Federation of Gynecology and Obstetrics [FIGO] 2009 staging system) SCC or adenocarcinoma following conization were included in the study. Patients were excluded if they had no pathological material to review, received cervical excision before conization, had positive LVSI or had other types of carcinoma. Medical records were reviewed for demographic data, treatment history and pathologic findings.

The primary outcome of the current study was a combined endpoint of recurrence and new-onset HSILs or invasive cancer (HSILs +) of the lower genital tract (cervix, vagina and vulva) 12 months or longer after initial conization. A ≥ 12-month interval was selected to offer the patients an opportunity for a second surgical intervention. Secondary endpoints, which were evaluated 12 months within the last follow-up, included high-risk HPV (hrHPV) infection and atypical squamous cells of undetermined significance (ASCUS) or worse (ASCUS +) on cytology. Based on the estimation of a meta-analysis^8^, we assumed that the 5-year accident rates of the primary outcome event of recurrence or new-onset HSIL+ were 10% and 5% in patients accepting conization and hysterectomy, respectively, and each group needed 85 participants to achieve a class I error probability (α value) of 0.05 and a statistical power of 0.8 in a noninferiority analysis with a noninferiority margin of 5% in conization patients. Considering loss to follow-up rate of approximately 10%, at least 189 patients should be included to accomplish the study goal.

### Intervention and follow-up

A cone-shaped portion of the cervix was excised to remove the cervical lesion and the entire transformation zone. A cone height of at least 10 mm was required for each patient. The specimen size was determined according to the pathological reports. Cold knife conization (CKC) and electrosurgical conization (ESC) have been described in a previous report^19^ and were performed depending on the surgeon’s preference with adequate estimation of the potential margin status. Because quick unipolar electronic cutting was utilized for resection, no heat denaturation occurred in the current study. When a lesion was suspected in the vagina or vulva, a biopsy was conducted at the same time.

The first follow-up occurred 6 weeks after the surgery, which provided an opportunity to discuss pathologic findings. Subsequent management, which included repeat conization, simple hysterectomy, and a follow-up, were provided and discussed with the patients. For patients desiring pregnancy, regular intercourse was encouraged 3 months after conization. In the first year after surgery, a follow-up was provided at an interval of 3 months; in the second year, a follow-up was provided at an interval of 6 to 12 months; and thereafter, a follow-up was provided yearly. The follow-up consisted of a pelvic examination, testing for hrHPV and cytology. The management of any abnormal findings followed current guidelines^20^. All patients were followed up until July 1, 2019. Six of 9 patients with IA1 SCC had a positive margin of HSIL, and another 3 patients had SCC lesions close to the margin. After comprehensive discussions, these nine patients denied further treatment and requested a long-term follow-up.

Obstetric outcomes were collected by medical records and/or patient interviews. The obstetric outcomes consisted of conception proven by human chorionic gonadotropin in the serum or urine or an ultrasonographic examination, and miscarriage or live birth was confirmed by medical/surgical records or a birth certificate. Live births included preterm and term births.

### Pathological assessment

The pathological assessment was described in a previous report^19^. Margin status, endocervical glandular involvement, invasion depth and width, and LVSI were evaluated. Any patient with missing or ambiguous information was reviewed by two pathologists (HW and YB). The diagnosis of carcinoma in situ and invasive cancer before 2010 was also reviewed and modified according to the FIGO 2009 staging system. Margin status (endocervical, ectocervical) was defined as positive based on the presence of AIS, invasive carcinoma or less than 1 mm at the edge of the specimens. Endocervical glandular involvement was defined as a dysplastic squamous epithelium occupying well-circumscribed, rounded spaces in the depth of the cervical stroma^21^.

### Statistical analysis

Comparisons of continuous variables were conducted using parametric methods if assumptions of a normal distribution were confirmed. Nonnormally distributed variables and categorical data were compared using nonparametric tests. The Cox regression method was used to analyze risk factors associated with the primary and secondary outcomes. The life-table method was used to calculate the cumulative conception and live birth rates. The odds ratios (ORs) or hazard ratios (HRs) with the 95% confidence intervals (95% CIs) were obtained from a multivariate model using significant clinicopathological factors. The data of patients lost to follow-up were treated as censored data. A receiver operating characteristic (ROC) curve was used to describe the association between age and obstetric outcomes. Unless otherwise stated, all analyses were performed with a two-sided significance level of 0.05 using SPSS 22.0 (SPSS, Inc., Chicago, IL, USA).

### Consent for publication

Consent for publication was obtained from all patients.

### Ethics approval and registration

The Institutional Review Board of Peking Union Medical College Hospital approved this study (No. S-K777). The registration number is NCT03961178 (*clinicaltrials.gov*). Written informed consent was obtained from all the participants in the study.

## Results

### Demographic data of the study population

**Figure 1:**
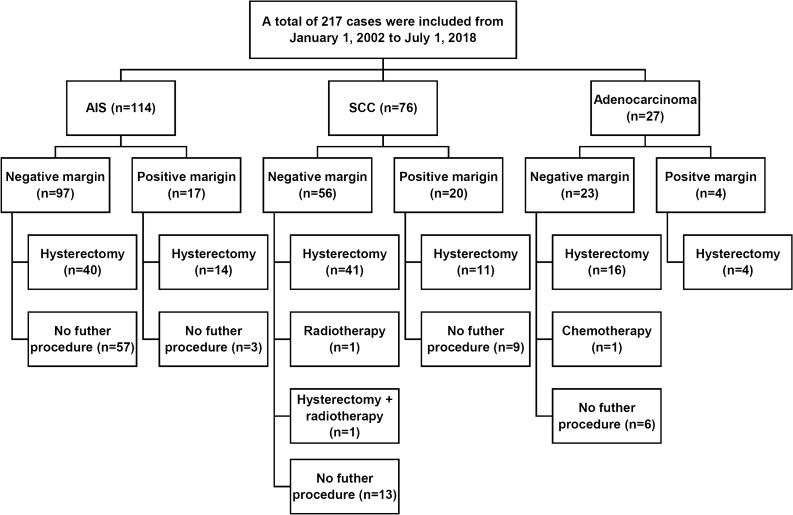
Pathological results and subsequent management in this study. One patient with adenocarcinoma requested chemotherapy because of enlarged pelvic lymph nodes found during hysterectomy. One patient with squamous cell carcinoma accepted radiotherapy due to suspicious lymphovascular space invasion; however, pathological review yielded negative findings.

The study flow diagram is illustrated in Fig. 1. The demographic characteristics of all patients and patients with AIS, SCC, and ADC are listed in Table 1. This retrospective study enrolled 217 patients, including 114 (52.5%) with AIS, 76 (35%) with SCC and 27 (12.4%) with ADC (Fig. 1). In the total series, the median patient age was 40 years (range, 24–71 years); 184 (84.8%) patients were premenopausal, and 53 (24.3%) were nulliparous. Among the patients, 184 (84.8%) received CKC, and 33 (15.2%) underwent ESC. Pathologic findings of the conization specimens showed that 76 (66.7%) AIS cases were accompanied by squamous cell dysplasia, including 5 (4.4%) with a low-grade squamous intraepithelial lesion (LSIL) and 71 (62.3%) with an HSIL. Eight cases (10.5%) of SCC were accompanied by AIS. LSILs and HSILs were also discovered in 17 (63%) and 4 (14.8%) ADC patients, respectively (Table 1). The median depth and width of the cone were 16 mm (rage 10–32) and 27 mm (range 14–42), respectively. The median depth and width of the cancer lesion were 1.5 mm (range, 1–3 mm) and 1 mm (range, 1–7 mm), respectively. For the SCC and invasive ADC groups, the median invasive width and depth had no significances (2 mm [range 1–7] vs 1 mm [1–7] for width, € *p* = 0.176; 2 mm [range 1–3] vs 1 mm [1–3] for depth, *p* = 0.500). The clinicopathological findings are summarized in Table 1.

**Table 1:** Clinicopathological features of the study populations. AIS, adenocarcinoma in situ. CKC, cold knife conization. ESC, electrosurgical conization. HSIL, high-grade squamous intraepithelial lesion. LSIL, low-grade squamous intraepithelial lesion. SCC, squamous cervical cancer.

	All patients (n = 217)	AIS (n = 114)	SCC (n = 76)	Invasive adenocarcinoma (n = 27)
Age (years), median (range)	39.85 (24–71)	37.71 (24–71)	40.46 (26–67)	41.13 (30–69)
Premenopause, n (%)	184 (84.8%)	99 (86.6%)	64 (84.2%)	21 (77.8%)
Initial conization				
CKC, n (%)	184 (84.8%)	91 (79.8%)	69 (90.8%)	24 (88.9%)
ESC, n (%)	33 (15.2%)	23 (20.2%)	7 (9.2%)	3 (11.1%)
Accompanied by other lesions, n (%)				
None	–	38 (33.3%)	68 (89.5%)	15 (55.6%)
AIS	–	–	8 (10.5%)	0 (0.0%)
LSIL	–	5 (4.4%)	0 (0.0%)	10 (37%)
HSIL	–	71 (62.3%)	0 (0.0%)	2 (7.4%)
Tumor differentiation, n (%)				
Well	13 (6%)	–	5 (6.6%)	8 (29.6%)
Moderately	12 (5.5%)	–	10 (13.2%)	2 (7.4%)
Poorly	4 (1.8%)	–	4 (5.3%)	N/A
Unknown	188 (86.6%)	–	57 (75%)	17 (63%)
Margin involvement, n (%)	41 (18.9%)	17 (14.9%)	20 (26.3%)	4 (14.8%)
Glandular involvement, n (%)	118 (54.4%)	56 (49.1%)	55 (72.4%)	7 (25.9%)
Invasion depth of cancer (mm), range (medium)	1.5 (1–3)	–	2 (1–3)	1 (1–3)
Invasion width of cancer (mm), range (medium)	1 (1–7)	–	2 (1–7)	1 (1–7)

In total, within 12 months after conization, 128 patients had their uterus removed by hysterectomy (117 cases) or radical hysterectomy (11 cases). All 11 radical hysterectomy procedures were performed for suspected stage IA2 disease due to a positive margin status in ADC or SCC patients. However, pathological reviews confirmed stage IA1 disease. In the AIS, SCC and ADC groups, the median interval from conization to hysterectomy was 56 (1–243) days, 41 (1–204) days and 41 (1–204) days, respectively. Two patients with SCC underwent radiotherapy and hysterectomy following radiotherapy.

### Margin involvement and its risk factors

Clinicopathological factors associated with margin involvement are listed in Table 2. Positive surgical margins were found in 41 (18.9%) patients, including 17 (14.9%) with AIS, 20 (26.3%) with SCC and 4 (14.8%) with adenocarcinoma. Two of 17 positive resection margins for AIS were HSILs, and the other 15 were AIS. The rates of endocervical and ectocervical involvement in AIS were 1.8% and 13.2%, respectively. For SCC, 70.0% of positive resection margins were squamous cancer, and the other 30.0% were HSILs. Among the 76 patients with SCC, endocervical, ectocervical, and both endocervical and ectocervical involvement occurred in 6 patients (7.9%), 13 patients (17.1%) and 1 patient (1.3%), respectively. In the ADC group, 4 positive resection margins were all adenocarcinoma; 1, 2 and 1 patients had endocervical, ectocervical and both endocervical and ectocervical involvement, respectively.

**Table 2:** Clinicopathological factors associated with margin involvement. AIS, adenocarcinoma in situ. CKC, cold knife conization. ESC, electrosurgical conization. N/A, not available. SCC, squamous cervical cancer.

	AIS margin status (n = 114)			SCC margin status (n = 76)			Adenocarcinoma margin status (n = 27)			All margin status (n = 217)		
	Negative	Positive	*p*	Negative	Positive	*p*	Negative	Positive	*p*	Negative	Positive	*p*
Age (years), medium (range)	37.05 (24–59)	38.13 (32–71)	0.124	40.46 (26–67)	40.97 (30–60)	0.972	40.44 (30–69)	43.42 (34–50)	0.585	34.91 (24–69)	41.28 (30–71)	0.252
Menopause, n (%)			0.696			0.500			0.545			0.551
No	85 (74.6%)	14 (12.3%)		46 (60.5%)	18 (23.7%)		17 (63%)	4 (14.8%)		148 (68.2%)	36 (16.6%)	
Yes	12 (10.5%)	3 (2.6%)		10 (13.2%)	2 (2.6%)		6 (22.2%)	0		28 (12.9%)	5 (2.3%)	
Conization method, n (%)			0.107			1.000			1.000			0.394
CKC	80 (70.2%)	11 (9.6%)		51 (67.1%)	18 (23.7%)		20 (74.1%)	4 (14.8%)		151 (69.6%)	33 (15.2%)	
ESC	17 (14.9%)	6 (5.3%)		5 (6.6%)	2 (2.6%)		3 (11.1%)	0		25 (11.5%)	8 (3.7%)	
Depth of the lesion (mm), medium (range)	N/A	N/A	N/A	2 (1–3)	1.75 (1–3)	0.983	1 (1–3)	2 (1–2)	0.650	1.5 (1–3)	2 (1–3)	0.835
Width of the lesion (mm), medium (range)	N/A	N/A	N/A	2 (1–7)	1 (1–7)	0.429	1 (1–7)	1 (1–6)	0.711	1 (1–7)	1 (1–7)	0.717
Glandular involvement, n (%)			0.216			0.374			0.545			0.918
No	47 (41.2%)	11 (9.6%)		17 (22.4%)	4 (5.3%)		16 (59.3%)	4 (14.8%)		80 (36.9%)	19 (8.8%)	
Yes	50 (43.9%)	6 (5.3%)		39 (51.3%)	16 (21.1%)		7 (25.9%)	0		96 (44.2%)	22 (10.1%)	
Accompanied by other lesions, n (%)	67 (58.8%)	9 (7.9%)	0.193	6 (7.9%)	2 (2.6%)	0.929	12 (44.4%)	0	0.106	85 (39.2%)	11 (5.1%)	0.013

In general, patients with positive and negative margins had similar average cone heights (15.0 ± 4.5 vs 16.6 ± 4.8 mm, respectively, *p* = 0.058) and similar average cone widths (26.9 ± 7.6 vs 27.0 ± 8.1 mm, respectively, *p* = 0.929). On univariate analysis, no risk factors were found to be associated with the involvement of the surgical margin in the AIS, SCC and ADC groups. However, in the whole cohort, only concurrent lesions were associated with a significantly high positive margin status (39.2% vs 5.1%, *p* = 0.013).

### Primary and secondary outcomes and risk factors

Fourteen (6.5%) patients were lost to follow-up beyond one year. For the remaining 203 patients, after a median follow-up of 36.5 months (range 12–207), 18 (8.9%) had histological examinations for abnormal follow-up results, and 5 (2.5%) were diagnosed with HSIL+ of the lower reproductive tract, one (0.5%) was diagnosed with a grade 1 vaginal squamous intraepithelial lesion (VAIN), and 12 had negative findings. Detailed information on the 5 patients who met the primary outcome of this study is displayed in Table 3. For the whole cohort, the cumulative incidence rates of HSILs + at 3, 5 and 10 years were 1.0%, 1.5% and 2.0%, respectively.

**Table 3:** Clinical features of five patients diagnosed with high-grade intraepithelial lesions or invasive cancer of the lower reproductive tract during the follow-up period. ADC, adenocarcinoma. AIS, adenocarcinoma in situ. CIN, cervical intraepithelial lesion. HSIL, high-grade squamous intraepithelial lesion. N/A, not available. SCC, squamous cervical cancer. RH, radical hysterectomy.

	Age	Menopause	Conization pathology	Margin involvement	Treatment after conization (days)	Ovary resection	Pregnancy	Diagnosis	Diagnosis after conization	Final treatment
Case 1	50	No	ADC	ADC (ectocervical)	Laparoscopic RH (44)	No	N/A	AIS in the vaginal stump	35 months	Radiotherapy
Case 2	48	No	SCC	Negative	Laparoscopic hysterectomy (49)	No	N/A	Minimally invasive squamous cancer in the vaginal stump	82 months	Radiotherapy
Case 3	34	No	SCC	HSIL (endocervical)	No further procedure	Yes	Not attempted	Squamous cancer in the vulva	33 months	Radiotherapy
Case 4	35	No	SCC	SCC (endo- and ectocervical)	No further procedure	Yes	Not attempted	CIN3	42 months	Hysterectomy
Case 5	37	No	SCC	SCC (endocervical)	No further procedure	Yes	Not attempted	SCC (FIGO Stage IA1)	174 months	RH

In patients accepting conization and hysterectomy, the rates of primary outcome events were 3.5% (3/86) and 1.7% (2/115). Based on the sample size estimation in the Methods section, this study achieved a statistic power of 0.9997.

After a median follow-up of 37 months (range 12–207), 42 (20.7%) patients had abnormal cytology (ASCUS +), and 57 (28.1%) patients had an hrHPV infection 12 months within the last follow-up. Among the cases of hrHPV infection, 33 and 22 cases had HPV 16/18 and other hrHPV subtypes, respectively. The cumulative incidence rates of ASCUS + at 3 and 5 years were 11.8% and 14.8%, respectively, and those of hrHPV were 14.8% and 19.7%, respectively.

In the whole cohort or subgroups based on various pathological results, hysterectomy had no significant impact on the occurrence of HSILs+ (Supplementary Table 1; Kaplan–Meier method). The results of the Cox regression analysis for primary and secondary outcomes are shown in Supplementary Table 2. No significant independent risk factors, including hysterectomy or margin status, were associated with HSILs + in the whole cohort or in various subgroups. In the whole cohort and in the SCC subgroup, age was the only risk factor associated with hrHPV infection (HR 1.12 and 1.12, 95% CI 1.04–1.21 and 1.02–1.23, *p* = 0.004 and 0.020, respectively). Margin involvement was the only risk factor associated with ASCUS + in the AIS group (HR 3.90, 95% CI 1.31–11.57, *p* = 0.014). Other concurrent lesions were independent of hrHPV infection in the AIS group (HR 2.62, 95% CI 1.01–6.81, *p* = 0.048).

### Pregnancy outcomes

**Figure 2:**
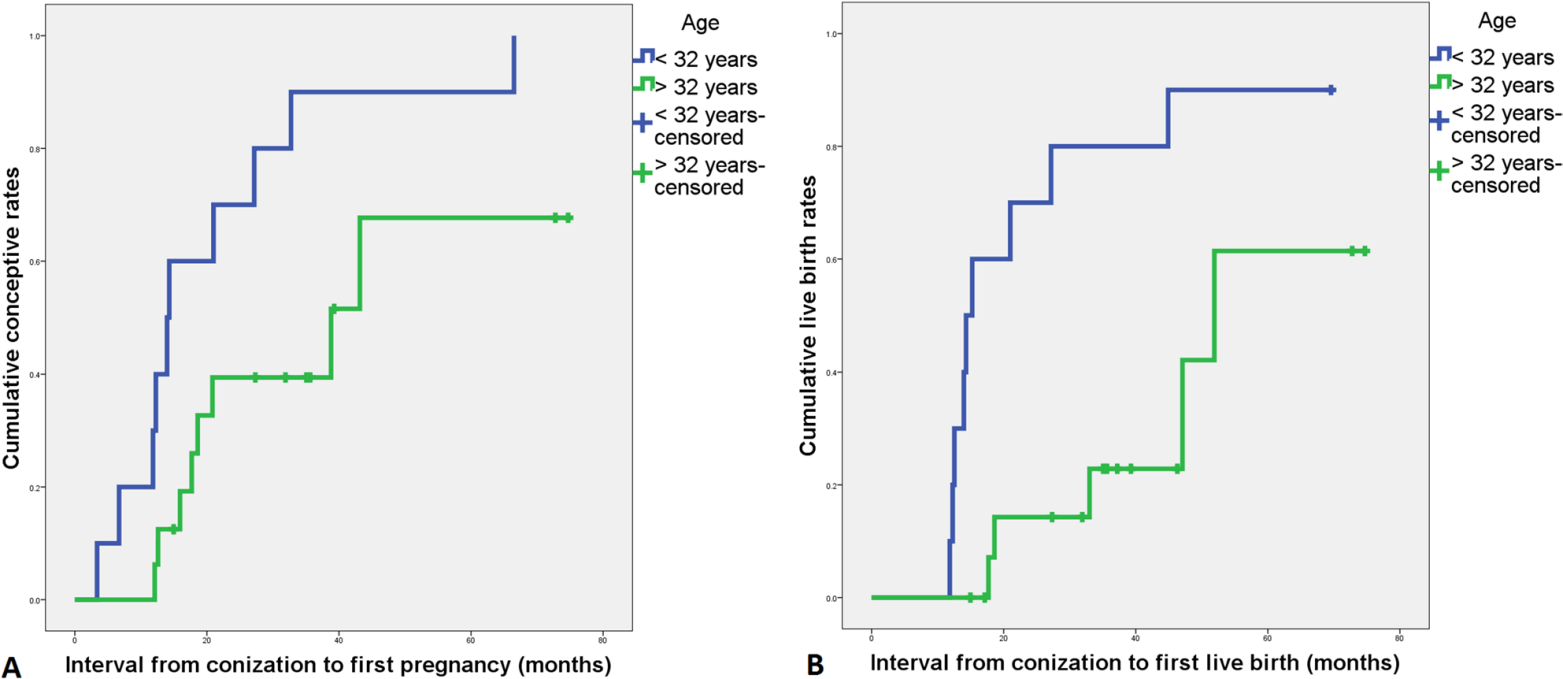
Cumulative pregnancy rate (**A**) and live birth rate (**B**) in patients < 32 years and > 32 years (Kaplan–Meier method).

The obstetric outcomes and risk factors are listed in Tables 4 and 5, respectively. Among the 88 patients with an intact uterus and no history of radiotherapy, 30 attempted conception. Their median age was 32 years (range 24–41). Five and 25 patients underwent ESC and CKC, respectively, and 25, 3, and 2 patients had AIS, stage IA1 SCC and ADC, respectively. As a result, 20 patients achieved 23 pregnancies, which resulted in 7 miscarriages and 16 live births. Among live births, 31.2% (5/16) were preterm births. In the whole cohort, the 3- and 5-year cumulative conception rates were 66% and 73%, respectively, the 3- and 5-year cumulative live birth rates were 48% and 71%. In the Cox regression model, age was the only independent risk factor for conception (HR 0.855, 95% CI 0.742–0.985, *p* = 0.031), live birth (HR 0.796, 95% CI 0.670–0.945, *p* = 0.009), and term birth (HR 0.826, 95% CI 0.711–0.960, *p* = 0.013) (Table 5). The areas under the ROC curves for successful conception and live birth were 0.735 (standard error 0.092, *p* = 0.039) and 0.786 (standard error 0.084, *p* = 0.008), respectively. The cumulative conception and live birth rates in patients < 32 years and > 32 years based on Kaplan–Meier methods are illustrated in Fig. 2.

**Table 4:** Pregnancy results after conization. ADC, invasive adenocarcinoma. AIS, adenocarcinoma in situ. SCC, squamous cell carcinoma.

	AIS group	SCC group	ADC group	All
Uterus intact (n)*	60	22	6	88
Attempting pregnancy (n)	25	3	2	30
Pregnancy (n)	17	2	1	20
Interval from conization to first pregnancy (months), median (range)	18 (3–75)	19 (7–51)	39	20 (3–75)
3-year cumulative pregnancy rates	65%	67%	50%	66%
5-year cumulative pregnancy rates	74%	67% at 48 months	50% at 39 months	73%
Abortion (n)	7	0	0	7
Live birth	13	2	1	16
Preterm (n)	0	1	1	2
Term labor (n)	13	1	0	11
Interval from conization to first live birth (months), median (range)	27 (12–75)	19 (15–51)	43 (39–47)	27 (12–75)
3-year cumulative live birth rates	50%	67%	0	48%
5-year cumulative live birth rates	69%	67% at 48 months	100% at 45 months	71%

*Patients with a radiotherapy history were excluded.

**Table 5:** Analysis of potential risk factors for pregnancy, live births and term births in patients attempting pregnancy. 95% CI, 95% confidence interval. ADC, invasive adenocarcinoma. AIS, adenocarcinoma in situ. CKC, cold knife conization. ESC, electrosurgical conization. HR, hazard ratio. N/A, not available. SCC, squamous cell carcinoma.

	Pregnancy		Live birth		Term birth	
	HR (95% CI)	*p*	HR (95% CI)	*p*	HR (95% CI)	p
Age at conization	0.855 (0.742–0.985)	**0.031**	0.796 (0.670–0.945)	**0.009**	0.826 (0.711–0.960)	**0.013**
Cervical lesions						
AIS	Reference	–	Reference	–	Reference	-
SCC	1.585 (0.190–13.220)	0.670	1.039 (0.156–6.934)	0.968	1.014 (0.147–7.005)	0.988
ADC	0.477 (0.057–4.028)	0.497	0.533 (0.060–4.732)	0.572	0.451 (0.052–3.903	0.469
Conization methods						
CKC	Reference	–	Reference	–	Reference	-
ESC	2.523 (0.619–10.275)	0.197	1.296 (0.142–11.843)	0.818	3.002 (0.694–12.984)	0.141
Margin status						
Negative	Reference	–	Reference	–	Reference	-
Positive	0.000 (0.000-N/A)	0.988	0.000 (0.000-N/A)	0.986	0.000 (0.000-N/A)	0.984
Glandular involvement						
No	Reference	-	Reference	-	Reference	-
Yes	0.270 (0.055–1.337)	0.109	0.207 (0.036–1.192)	0.078	0.287 (0.055–1.497)	0.138
Complicated with CIN1 +						
No	Reference	–	Reference	–	Reference	-
Yes	1.139 (0.211–6.150)	0.880	0.952 (0.146–6.217)	0.959	0.951 (0.164–5.508)	0.956

The bold figures are all < 0.05.

However, almost all patients who attempted conception except for one had a negative margin status. This patient was 37 years old, had a diagnosis of AIS with a positive margin, and did not achieve successful conception during a follow-up of 27 months.

In five patients with recurrent or new-onset HSILs + , three received fertility-sparing treatments, but none attempted conception since they each had two healthy children.

Patients with and without successful conception had similar average cone heights (14.8 ± 3.1 vs 14.4 ± 3.3 mm, respectively, *p* = 0.714) and similar average cone widths (23.4 ± 6.5 vs 28.5 ± 7.6 mm, respectively, *p* = 0.067). For 16 live births, patients with term and preterm births had similar average cone heights (15.6 ± 3.0 vs 13.2 ± 2.8 mm, respectively, *p* = 0.150) and similar average cone widths (22.7 ± 6.4 vs 25.0 ± 8.3 mm, respectively, *p* = 0.557).

## Discussion

The present study provided evidence demonstrating that fertility preservation is safe for women with AIS, stage IA1 adenocarcinoma or SCC. This population-based study found no differences in disease recurrence, new-onset HSILs or invasive cancer in the lower reproductive tract after initial treatment with CKC and ESC. There were also no differences in hrHPV infection or ASCUS+ of cytology evaluation with CKC and ESC during the follow-up periods. For patients with an intact uterus and those in whom the uterus was removed, no differences were identified regarding disease recurrences. Margin status had no significant influence on recurrence or new-onset HSILs. However, women diagnosed with CIN3 by conization have been reported to have a long-lasting increased risk of cervical cancer even when the margins on the cone were negative^22–24^. A long, meticulous follow-up is essential for early-stage cervical cancer patients who undergo fertility-sparing procedures.

Most patients (52.5%) in our study had AIS. The risk of AIS recurrence is reported to be 0% and 47% after conservative treatment^25–28^. The risk of residual disease in AIS was reported to be reduced when a disease-free margin of 10 mm was achieved^8,29^. In our study, only one patient with AIS (0.9%) with a positive margin status had a recurrent HSIL. For AIS patients, however, residual disease was found in 16.5% of specimens from repeated conization or hysterectomy, even after conization with negative margins^30^. One possible explanation for this finding may be that AIS is considered a multifocal disease and is located beyond the proximal end of the endocervical cutting edge of the cone^30^. An endocervical curettage specimen positive for AIS was associated with residual AIS in 95% of cases^31^. Progression to adenocarcinoma of the cervix after conization demonstrating AIS has also been described^32^. Possible explanations for this finding include an inconsistent pathology interpretation, persistent HPV infections, the presence of skip lesions, and/or a residual, undetected residual focus of AIS^32^. Therefore, effective surveillance, particularly HPV testing, is very important in patients with AIS after conservative treatment^33^.

Generally, patients with stage IA1 cervical cancer had favorable oncologic outcomes. After a median of follow-up of approximately three years, all patients were alive, 4 patients (2.0%) patients had recurrent cancer, and one patient (0.5%) had a new-onset HSIL (CIN3). Patients with stage IA1 SCC probably comprise the most appropriate population to receive fertility-sparing treatment. Four of five patients with recurrent HSILs + had SCC as an initial diagnosis, and three had a positive margin. A review of 19 studies showed that the recurrence rate was 1.1% with all types of therapy^15^. In previous studies, approximately 96% of patients with stage IA1 cervical cancer who were treated conservatively were found to be alive and disease-free after 5 years of follow-up, and the recurrence rate ranged from 2.7 to 9%^34,35^. Given the favorable prognosis of stage IA1 cervical cancer, conservative treatment has often been advocated, particularly for young women^36^. Repeat excision for CIN present at the excision margin may not be necessary as long as the invasive focus is fully excised^9,37^.

In our study, none of 27 stage IA1 ADC patients had recurrence of HSILs+. A review of 1223 patients focused on stage IA1 cervical ADC found that the recurrence rate was 2.4% and claimed that the conization procedure was safe for stage IA1 cervical cancer^38^. A meta-analysis showed that the progression-free survival (PFS) and overall survival (OS) rates of stage IA1 cervical ADC patients who underwent fertility-sparing management were 98.8% and 98.9%, respectively^39^. Patients with microinvasive ADC who meet the criteria for FIGO stage IA1 cervical carcinoma and have negative margins appear to be at no greater risk for persistent or recurrent disease than patients with AIS alone^40^. No significant difference in survival was noted when patients with microinvasive ADC were compared by cell type or surgical procedure, suggesting that survival is not improved by utilizing more invasive surgical methods^7^. All these results show that the prognosis of patients with microinvasive cervical ADC is excellent and that fertility preservation is safe for young women with stage IA1 ADC.

Data in our study also showed that age was a risk factor associated with hrHPV infection and abnormal cytology screening, which may be due to the migration of the transformation zone to the cervical canal with age and subsequent menopause^41^. Our results showed that the margin involvement rate in AIS was 14.9%, which was the only risk factor associated with abnormal cervical screening during the follow-up after initial treatments. Many potential reasons may account for persistent HPV infection after conization. In the study of Costa et al.^42^, age, lesion grade, length of an active sexual life, and the involvement of surgical margins significantly predicted HPV persistence. Viral load was also important in predicting HPV persistence^43^. Postoperative hrHPV infection was a significant positive predictor for the reappearance of abnormal cytology and HPV16 infection-induced HSILs after treatment^44^. Findings from these current studies suggest that the posttreatment follow-up should include both cytology and hrHPV testing for women of all ages to detect patients with an increased risk of disease recurrence.

Our study showed that obstetric outcomes were favorable, with cumulative 3-year conceptive and live birth rates exceeding 50%. In addition, we found that age at first conization had a significant impact on a successful conception, live births and term births. These findings support conization as a feasible procedure for populations with AIS and stage IA1 cervical cancer. However, almost all patients attempting pregnancy had a negative margin status, which interfered with the analysis of the impact of pathological characteristics on obstetric outcomes. Conization can lead to impaired obstetric outcomes. The incidence of preterm deliveries following conization varies between 14 and 25% according to previous reports^45,46^. The risk of preterm delivery and a late spontaneous miscarriage increased in direct proportion to the cone size^47^. Caution should be recommended in the treatment of young women with mild cervical abnormalities. Obstetric outcomes and gynecologic prognosis should be weighed and balanced when deciding on cone size. However, in our study, due to the limited sample size of live birth, no significant differences were found in the cone height and width between patients with term and preterm births. In our analysis, the energy methods (CKC vs ESC) had no significant impact on the obstetric outcomes, which proved the feasibility and safety of ESC.

The large sample size, comprehensive pathological description, and complete clinical and obstetric outcomes obtained by close follow-up were the main strengths of our study. One of the limitations to this study is the retrospective and observational nature of the data, which lack randomization. Another limitation was that some patients had a short-term follow-up, which may underestimate the rate of recurrence. A long-term follow-up and a larger sample size are required to confirm these conclusions.

## Conclusions

Our study suggests that conization is safe for young women with AIS, stage IA1 SCC and ADC who desire future fertility, especially patients with negative resection margins. No significant risk factors were found to be associated with recurrence or new-onset HSILs+ in the lower genital tract, including disease nature, hysterectomy or margin status. Patients who preserved their fertility had favorable obstetric outcomes, but older age affected conception and live birth rates, suggesting that these patients should attempt pregnancy as soon as possible after conization.

## Supplementary information

Supplementary Information 1.

Supplementary Information 2.

Supplementary Information 3.

## Data Availability

All data in this study are contained in the supplement file.

## Statement of submission

The paper is not under consideration by another journal, and the results presented in this work have not been previously presented or published.

## Study design

Retrospective cohort study.
