# Supplementary material for: Oncologic and obstetric outcomes after conization for adenocarcinoma in situ or stage IA1 cervical cancer
Source: Sci Rep. 2020 Nov 16;10:19920. doi: 10.1038/s41598-020-75512-9 (PMC7669853; doi:10.1038/s41598-020-75512-9)
Supplement: Supplementary file 1 — Supplementary Information 1. [file 41598_2020_75512_MOESM1_ESM.docx]

Supplementary Table 1

Comparisons of study outcomes in patients with an intact uterus and those in whom the uterus was removed. ADC, adenocarcinoma. AIS, adenocarcinoma in situ. SCC, squamous cervical cancer. ASCUS+, atypical squamous cells of undetermined significance or worse. hrHPV, high-risk human papillomavirus.

|  | Cancer/HSIL | | Abnormal cytology/hrHPV | | |
| --- | --- | --- | --- | --- | --- |
|  | Incidence, n/n (%) | Follow-up interval (months), median (range) | Incidence of ASCUS+, n/n (%) | Incidence of hrHPV infection, n/n (%) | Follow-up interval (months), median (range) |
| AIS group |  |  |  |  |  |
| Uterine intact | 0/59 (0.0%) | 24.8 (12-153) | 12/58 (20.7%) | 13/58 (22.4%) | 25.03 (12-153) |
| Uterine removed | 0/52 (0.0%) | 25.38 (12-125) | 11/53 (20.8%) | 17/53 (32.1%) | 25.33 (12-125) |
| *p* | 1.000 | 0.927 | 0.993 | 0.252 | 0.960 |
| SCC group |  |  |  |  |  |
| Uterine intact | 3/20 (15.0%) | 42.77 (12-174) | 2/16 (12.5%) | 5/16 (31.2%) | 41.05 (12-103) |
| Uterine removed | 1/44 (2.3%) | 68.03 (12-207) | 10/46 (21.7%) | 12/46 (26.1%) | 68.03 (12-207) |
| *p* | 0.087 | 0.213 | 0.714 | 0.750 | 0.122 |
| Invasive ADC group |  |  |  |  |  |
| Uterine intact | 0/7 (0.0) | 56.3 (14-127) | 2/7 (28.6%) | 2/7 (28.6%) | 56.3 (14-127) |
| Uterine removed | 1/19 (5.3%) | 41.1 (16-119) | 3/18 (16.7%) | 4/18 (22.2%) | 45.63 (16-119) |
| *p* | 1.000 | 0.544 | 0.597 | 1.000 | 0.586 |
| All |  |  |  |  |  |
| Uterine intact | 3/86 (3.5%) | 36.98 (12-174) | 16/81 (19.8%) | 20/81 (10.1%) | 35.52 (12-195) |
| Uterine removed | 2/115 (1.7%) | 38.47 (12-207) | 24/117 (12.1%) | 33/117 (16.7%) | 38.53 (12-207) |
| *p* | 0.431 | 0.102 | 0.896 | 0.583 | 0.066 |
