# Supplementary material for: Oncologic and obstetric outcomes after conization for adenocarcinoma in situ or stage IA1 cervical cancer
Source: Sci Rep. 2020 Nov 16;10:19920. doi: 10.1038/s41598-020-75512-9 (PMC7669853; doi:10.1038/s41598-020-75512-9)
Supplement: Supplementary file 2 — Supplementary Information 2. [file 41598_2020_75512_MOESM2_ESM.docx]

Supplementary Table 2

Clinicopathological factors associated with primary and secondary outcomes in the multivariate model. ADC, adenocarcinoma. AIS, adenocarcinoma in situ. SCC, squamous cervical cancer. HSIL, high-grade squamous intraepithelial lesion. ASCUS+, atypical squamous cells of undetermined significance or worse. hrHPV, high-risk human papillomavirus. N/A, not available.

|  | Age | Menopause | Margin involvement | Hysterectomy removed | Glandular involvement | Accompany by other lesions | Invasion depth of cancer (mm) | Invasion width of cancer (mm) |
| --- | --- | --- | --- | --- | --- | --- | --- | --- |
| AIS group* |  |  |  |  |  |  |  |  |
| ASCUS+ |  |  |  |  |  |  |  |  |
| HR (95% CI) | 1.01 (0.93-1.09) | 1.64 (0.28-9.49) | 3.90 (1.31-11.57) | 0.75 (0.29-1.96) | N/A | 2.74 (0.91-8.27) | N/A | N/A |
| p | 0.905 | 0.582 | **0.014** | 0.560 | N/A | 0.073 | N/A | N/A |
| HrHPV |  |  |  |  |  |  |  |  |
| HR (95% CI) | 1.00 (0.93-1.08) | 1.60 (0.35-7.32) | 2.71 (0.99-7.37) | 1.06 (0.47-2.40) | N/A | 2.62 (1.01-6.81) | N/A | N/A |
| p | 0.973 | 0.545 | 0.052 | 0.895 | N/A | **0.048** | N/A | N/A |
| SCC group |  |  |  |  |  |  |  |  |
| ASCUS+ |  |  |  |  |  |  |  |  |
| HR (95% CI) | 1.17 (0.98-1.28) | 0.49 (0.40-6.06) | 1.16 (0.27-5.05) | 2.38 (0.27-21.14) | 2.02 (0.32-12.92) | 0.86 (0.08-9.66) | 0.54 (0.17-1.71) | 1.16 (0.74-1.82) |
| p | 0.106 | 0.581 | 0.841 | 0.435 | 0.457 | 0.899 | 0.296 | 0.506 |
| HrHPV |  |  |  |  |  |  |  |  |
| HR (95% CI) | 1.12 (1.02-1.23) | 0.55 (0.09-3.33) | 1.20 (0.40-3.61) | 0.40 (0.12-1.29) | 1.09 (0.32-3.77) | 0.80 (0.08-7.84) | 0.46 (0.19-1.13) | 1.23 (0.87-1.75) |
| p | **0.020** | 0.517 | 0.746 | 0.124 | 0.892 | 0.850 | 0.089 | 0.248 |
| Cancer/HSIL |  |  |  |  |  |  |  |  |
| HR (95% CI) | 1.22 (0.69-2.15) | N/A | 107 (0.11-108179) | 0.87 (0.01-79.99) | 11707 (N/A) | 116.66 (N/A) | 0.03 (0-2.38) | 0 (N/A) |
| p | 0.501 | 0.979 | 0.185 | 0.951 | 0.961 | 0.996 | 0.117 | 0.882 |
| ADC group |  |  |  |  |  |  |  |  |
| ASCUS+ |  |  |  |  |  |  |  |  |
| HR (95% CI) | 1.01 (0.73-1.40) | 5552.84 (N/A) | 12132.71 (N/A) | 0 (N/A) | 0 (N/A) | 0 (N/A) | 1.37 (0.37-5.02) | 0.63 (0.24-1.65) |
| p | 0.946 | 0.969 | 0.966 | 0.965 | 0.964 | 0.970 | 0.637 | 0.349 |
| HrHPV |  |  |  |  |  |  |  |  |
| HR (95% CI) | 1.24 (0.93-1.65) | 0.093 (0-14.84) | 0.137 (0-9.21) | 0.92 (0.03-28.48) | 0.69 (0.01-42.44) | 0.22 (0.01-10.03) | 1.37 (0.30-6.21) | 0.92 (1.44-1.92) |
| p | 0.152 | 0.359 | 0.354 | 0.960 | 0.861 | 0.440 | 0.683 | 0.816 |
| Cancer/HSIL |  |  |  |  |  |  |  |  |
| HR (95% CI) | 1.75 (N/A) | 0 (N/A) | 85.92 (N/A) | 0.17 (N/A) | 0.01 (N/A) | 14.65 (N/A) | 0.15 (N/A) | 1.05 (N/A) |
| p | 0.946 | 0.975 | 0.984 | 0.994 | 0.983 | 0.991 | 0.968 | 0.998 |
| All patients |  |  |  |  |  |  |  |  |
| ASCUS+ |  |  |  |  |  |  |  |  |
| HR (95% CI) | 1.10 (1.00-1.21) | 0.37 (0.05-2.51) | 1.54 (0.48-4.95) | 1.56 (0.40-6.07) | 0.93 (0.31-52.80) | 1.35 (0.34-5.31) | 0.70 (0.31-1.55) | 1.00 (0.72-1.38) |
| p | 0.055 | 0.305 | 0.465 | 0.522 | 0.901 | 0.672 | 0.375 | 0.986 |
| HrHPV |  |  |  |  |  |  |  |  |
| HR (95% CI) | 1.12 (1.04-1.21) | 0.61 (0.14-2.72) | 1.06 (0.41-2.75) | 0.42 (0.16-1.09) | 0.97 (0.39-2.40) | 0.69 (0.19-2.57) | 0.54 (0.27-1.10) | 1.14 (0.86-1.52) |
| p | **0.004** | 0.518 | 0.904 | 0.074 | 0.943 | 0.581 | 0.089 | 0.367 |
| Cancer/HSIL |  |  |  |  |  |  |  |  |
| HR (95% CI) | 1.26 (0.76-2.10) | 0 (N/A) | 199.4 (0.43-92567) | 0.98 (0.01-146.9) | 17.28 (0.03-10885) | 0 (N/A) | 0.02 (0-1.56) | 0 (N/A) |
| p | 0.373 | 0.972 | 0.091 | 0.995 | 0.386 | 0.985 | 0.079 | 0.851 |

* HSIL+ occurred in no case in the group of ADC in situ, hence no statistical analysis was performed about the HR of HSIL+.
